# The association of marital/partner status with patient-reported health outcomes following acute myocardial infarction or stroke: Protocol for a systematic review and meta-analysis

**DOI:** 10.1371/journal.pone.0267771

**Published:** 2022-11-15

**Authors:** Cenjing Zhu, Phoebe M. Tran, Erica C. Leifheit, Erica S. Spatz, Rachel P. Dreyer, Kate Nyhan, Shi-Yi Wang, Larry B. Goldstein, Judith H. Lichtman

**Affiliations:** 1 Department of Chronic Disease Epidemiology, Yale School of Public Health, Yale University, New Haven, CT, United States of America; 2 Center for Outcomes Research and Evaluation, Yale University School of Medicine, New Haven, CT, United States of America; 3 Department of Internal Medicine, Section of Cardiovascular Medicine, Yale University School of Medicine, New Haven, CT, United States of America; 4 Department of Biostatistics, Yale School of Public Health, Yale University, New Haven, CT, United States of America; 5 Department of Emergency Medicine, Yale School of Medicine, New Haven, CT, United States of America; 6 Harvey Cushing/John Hay Whitney Medical Library, Yale University, New Haven, CT, United States of America; 7 Environmental Health Sciences, Yale School of Public Health, Yale University, New Haven, CT, United States of America; 8 Department of Neurology, University of Kentucky, Lexington, KY, United States of America; Tehran University of Medical Sciences, ISLAMIC REPUBLIC OF IRAN

## Abstract

**Introduction:**

Marital/Partner support is associated with lower mortality and morbidity following acute myocardial infarction (AMI) and stroke. Despite an increasing focus on the effect of patient-centered factors on health outcomes, little is known about the impact of marital/partner status on patient-reported outcome measures (PROMs).

**Objective:**

To synthesize evidence of the association between marital/partner status and PROMs after AMI and stroke and to determine whether associations differ by sex.

**Methods and analysis:**

We will search MEDLINE (via Ovid), Web of Science Core Collection (as licensed by Yale University), Scopus, EMBASE (via Ovid), and PsycINFO (via Ovid) from inception to July 15, 2022. Two authors will independently screen titles, abstracts, and then full texts as appropriate, extract data, and assess risk of bias. Conflicts will be resolved by discussion with a third reviewer. The primary outcomes will be the associations between marital/partner status and PROMs. An outcome framework was designed to classify PROMs into four domains (health-related quality of life, functional status, symptoms, and personal recovery). Meta-analysis will be conducted if appropriate. Subgroup analysis by sex and meta-regression with a covariate for the proportion of male participants will be performed to explore differences by sex.

**Ethics and dissemination:**

This research is exempt from ethics approval because the study will be conducted using published data. We will disseminate the results of the analysis in a related peer-reviewed journal.

**Trial registration:**

**PROSPERO registration number:** CRD42022295975.

## Introduction

Marital/Partner status is an important social factor that affects acute myocardial infarction (AMI) and stroke outcomes. Being married/partnered has been associated with lower mortality and higher event-free survival following an AMI or stroke, supported by studies varying in design, setting, and scale [1–6]. However, little is known about the impact of marital/partner status on patient-centered outcomes.

Patient-reported outcome measures (PROMs) are defined by the Food & Drug Administration and National Quality Forum as outcomes that derive directly from the patient about the status of a patient’s health condition without amendment or interpretation of the patient’s response by a clinician [7, 8]. With strides in cardiovascular disease and stroke, numerous PROMs have been developed, validated, and used in clinical studies to quantify treatment benefits with regard to improvements in symptoms, functional outcomes, and health-related quality of life (HRQoL) [9–11]. These measures also independently predict subsequent cardiovascular events, hospitalizations, costs of care, and mortality, and they have the potential to inform clinical decision making and targets for risk adjustment [12].

Prior studies assessed patient-reported health status outcomes among AMI and stroke patients using different PROMs, but the results regarding the association between marital/partner status and outcomes are inconsistent [13–16]. No systematic review investigates and synthesizes the association of marital/partner status with PROMs among individuals who have had an AMI or stroke. Further, although women may not benefit from marriage to the same extent as men regarding outcomes such as mortality and major adverse cardiac events [2, 17, 18], less is known about whether there are sex differences in the degree of “protection” conferred by marriage or partnership during AMI and stroke recovery as assessed with PROMs.

## Objective

The purpose of this report is to establish the explicit methodology we will use for conducting a systematic review and meta-analysis to answer the following questions: 1, does marital/partner status impact the patient-reported health status outcomes of individuals who have had an AMI or stroke; and 2, are there sex differences in the impact of marital/partner status on patient-reported health status outcomes of individuals who had an AMI or stroke?

## Methods

**Fig 1:**
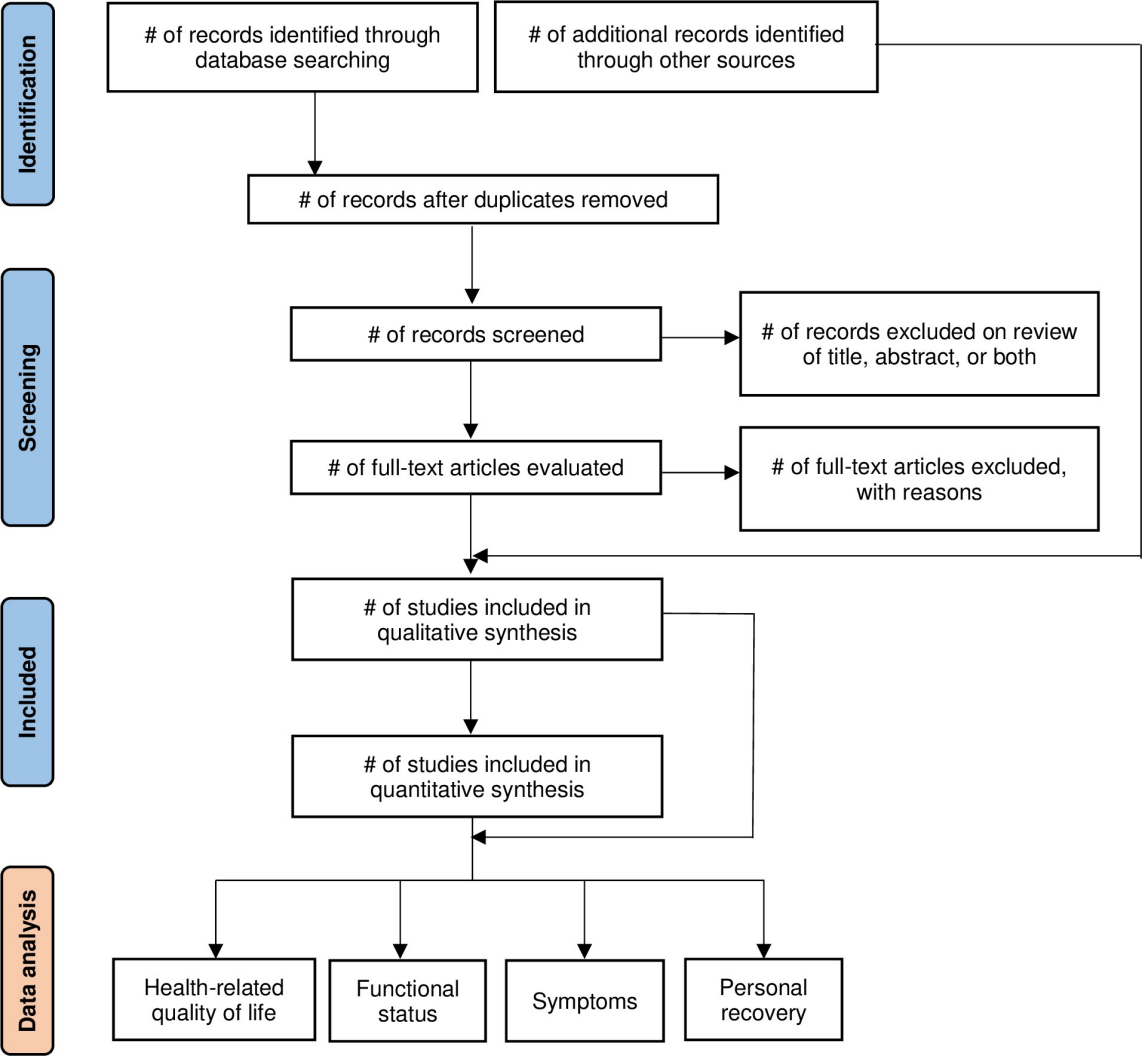
Flowchart of literature search, selection, and analysis.

The review protocol complies with the Preferred Reporting Items for Systematic Reviews and Meta-Analyses Protocols (PRISMA-P) statement guidance [19] for reporting the present protocol and subsequent formal meta-analysis. The review protocol is registered on the International Prospective Register of Systematic Reviews (PROSPERO) database (ID: CRD42022295975). An overview of the literature search and analysis process is given in **Fig 1**.

### Search strategy and information sources

We will conduct a literature search using Medline (via Ovid), Web of Science Core Collection (as licensed by Yale University), Scopus, EMBASE (via Ovid), and PsycINFO (via Ovid) to identify publications from inception to July 15, 2022. **Table 1** displays the participants, exposure, comparison, and outcome (PECO) criteria, concepts, and search strategy for the planned review. For each concept in the search strategy, we use appropriate keywords and subject indexing terms, informed by the search strategies used in previous reports. The search strategy was developed and adapted for individual databases and interfaces as needed by the first author in consultation with a librarian with expertise in medical science and literature searches (details in **S1 Table**). Studies on AMI and stroke were searched and will be reviewed separately. For each included article that meets the eligibility criteria, we will conduct forward citation chaining in The Lens via Citation Chaser [20].

**Table 1:** PECO elements, concepts, and keywords.

Element	MeSH	
**Population:** Adults with AMI OR Adults with stroke	myocardial infarction, acute coronary syndrome, coronary disease	coronary heart disease, ischemic/ischaemic heart disease, heart attack, AMI, myocardial infarct
	stroke, cerebrovascular disorders	cerebrovascular disease, cerebrovascular accident, brain vascular accident
**Exposure:** Marital/Partner status	marital status (narrower terms: “divorce”, “marriage”, “spouses”, “marital status”, “single person”, “widowhood”)	marital/spousal relationship, unmarried, partnership, marriage, wife/husband, spouse, domestic partner, break-up, divorce*, widow*, single, spinster, bachelor, girlfriend, boyfriend
		Notes: We will only consider papers that assess marital/relationship status as an independent exposure. We will not consider a composite exposure that includes marital status (e.g., ‘cohabitation’ if defined based on both marital status and living condition; or living with spouse or family members).
**Comparison:**	Married/Partnered vs unpartnered	
**Outcome:** Patient-reported outcome measures	patient reported outcome measures (2017); patient outcome assessment (2013–2016)	patient-reported outcome*, self-report*, patient-reported*, patient-centered*, preference, experience, perception, perceived, measure*, questionnaire*, survey*, psychometric*, depress*, psychologic*, anxiety, symptom*, fatigue
		Notes: We combined 3 approaches to identify PROMs:
		1. Oxford filter for PROM: https://cosmin.nl/wp-content/uploads/prom-search-filter-oxford-2010.pdf (available for Ovid and PubMed, translated to Web of Science and Scopus by C.Z.)
		2. MeSH ("Patient Reported Outcome Measures"; "Patient Outcome Assessment") + keywords search for “patient-reported health status”
		3. Identified from literature, scoping search (via ResearchRabbit), and NINDS CDE, most commonly used PROMs in AMI/stroke:
		• Generic: EQ-5D, Short Form-12/36, General Health Questionnaire, PROMIS • Stroke-specific: Neuro-QOL, Stroke Impact Scale, Stroke Specific QOL, SAQOL, SA-SIP • AMI-specific: QLMI, Seattle Angina Questionnaire, Mac-New Questionnaire, HeartQoL, QLICD_CHD

### Eligibility criteria

The systematic review will identify studies that evaluate marital/partner status as an independent variable and report their associations with one or more defined PROMs. The included studies should have at least two groups (married vs. unmarried, or partnered vs. not partnered). Studies with no clearly indicated reference/control group or defined outcome will be excluded. Study participants will include individuals aged 18 years and older who were diagnosed with an AMI or stroke by a medical professional. PROMs may be obtained from proxy respondents if the patient was not able to answer. Animal studies will not be included. No restrictions will be placed on study type, setting, time frame, or publication year. Only reports that are available in print or downloadable form and written in English will be included.

### Data extraction and management

Records identified from the database search will be uploaded into a Covidence project. After deduplication, unique records will be screened independently at the title-abstract stage by two reviewers (C.Z. and P.T.) using pre-specified inclusion criteria. The full text of the identified studies will then be assessed by the two reviewers, with documentation of the reasons for any exclusions. A third researcher will help the screeners achieve consensus in cases of disagreement.

After screening, two researchers will independently extract the data on author information, publication year, country, study type (observational vs. interventional), study setting (institutionalized vs. community-dwelling), features of the study participants, the main exposure definition(s) (married/partnered vs. unmarried/not partnered or divorced/separated, widowed), the main outcome definition(s) (PROM, name, type, measurement domain, time points), the number of individuals with the outcome based on the exposure, the effect estimates (adjusted odds ratios, risks ratios, hazard ratios, or regression coefficients [β] with standard errors [SEs], which can be calculated from 95% confidence intervals [CIs] or p-values) and adjustment factors, sex-specific estimates (if reported), and disease type (AMI or stroke).

The primary outcome of the review is the association between marital/partner status and PROMs. Relevant data will be recorded using a standardized data extraction form (**S2 Table**). Authors of the included studies will be contacted for missing key information, including marital/partner status reference group, PROM definitions, and results of the association analyses (i.e., effect estimates, SE, and significance).

### Quality assessment

Methodological quality (risk of bias) of the included studies will be appraised using the Newcastle-Ottawa Scale [21, 22]. This scale is the most commonly used methodological quality assessment measure for observational studies. The scale output will be converted to the Agency for Healthcare Research and Quality standard (good, fair, poor quality) based on previously defined thresholds [22]. The quality of the PROMs (i.e., their measurement properties) identified from all included studies will be evaluated using the COnsensus-based Standards for the selection of health Measurement INstruments (COSMIN) risk of bias checklist [23–25].

### Data synthesis and subgroup analysis

**Fig 2:**
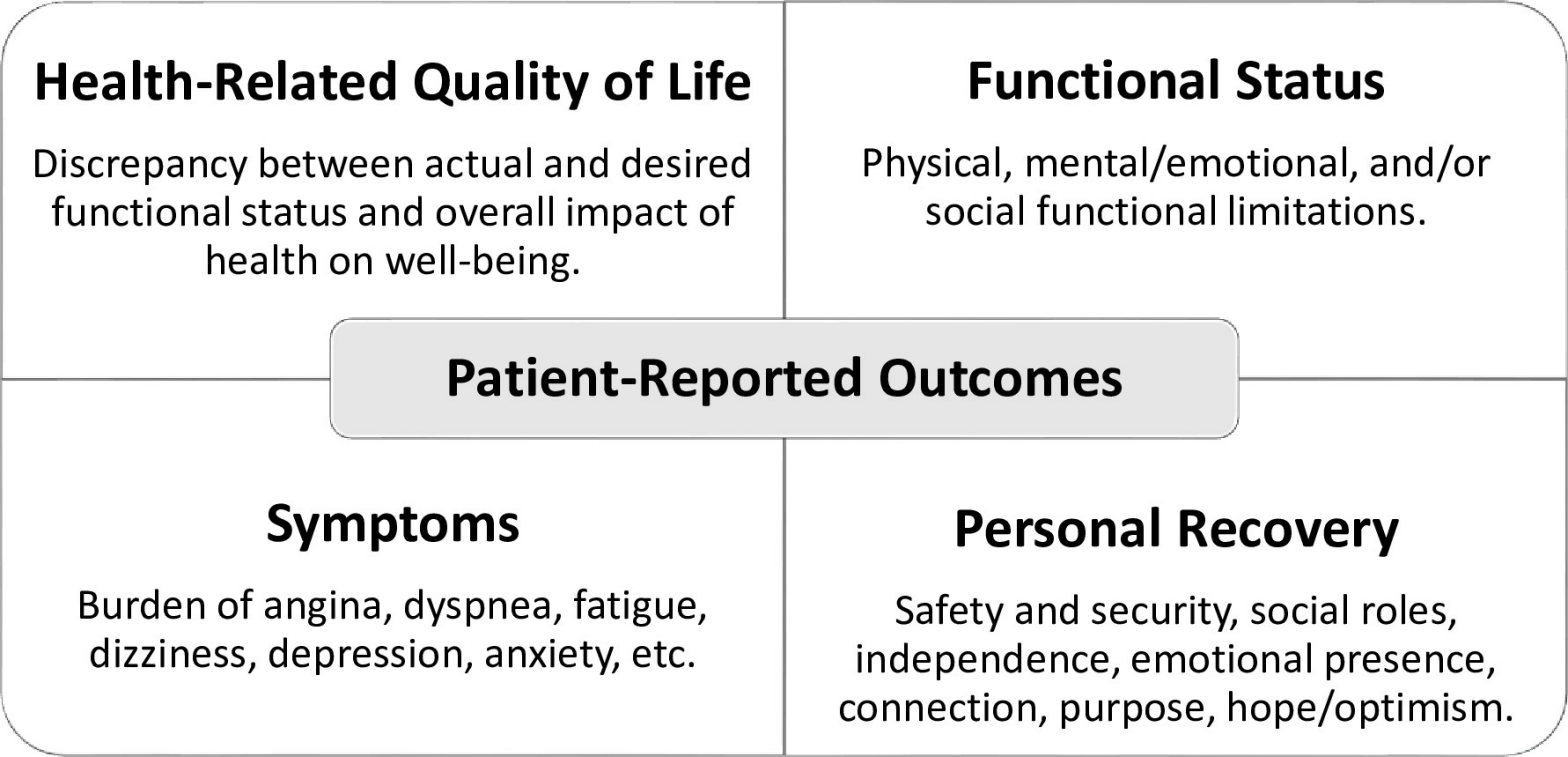
PROM domains and constructs.

An outcome framework (**Fig 2**) based on American Heart Association statement papers and prior research [9, 11, 26] was designed to classify PROM outcomes. We started from the patient-reported outcome taxonomy of Rumsfeld et al. and Reeves et al. for AMI and stroke, respectively, in which PROMs were classified into three domains (HRQoL, functional status, and symptoms) [9, 11]. To capture outcomes from a bio-psycho-social perspective, we further added a “personal recovery” outcome domain based on a conceptual framework of patient-centered recovery in cardiology proposed by Dreyer et al., [26] which is also supported by stroke literature [27]. Personal recovery refers to a multidimensional process—“a deeply personal, unique process of changing one’s attitudes, values, feeling, goals, skills and/or roles.” Examples of personal recovery outcomes are competence, empowerment, well-being, and activation. Results from the included studies will be classified into the outcome framework, and data synthesis will be carried out accordingly.

After classification, we will first determine the feasibility of conducting a meta-analysis. Meta-analyses will be conducted if there are three or more studies reporting effect measures and 95% CIs for the same PROM, or if there are three or more different PROMs measuring a specific construct. In the cases in which a particular outcome construct was measured by different PROMs (e.g., Seattle Angina Questionnaire, SF-12, EQ-5D to measure HRQoL), the two reviewers will compare the content of the PROMs, assess their measurement properties (i.e., reliability and validity), and decide whether statistical pooling is appropriate (i.e., if the features of these studies are sufficiently similar to combine). If conducting a meta-analysis is feasible and appropriate, effect sizes will be converted into standardized mean differences and combined using a random-effects model with inverse-variance weighting to calculate pooled effect sizes. For studies reporting both unadjusted and adjusted effect estimates, the adjusted effect judged to minimize the risk of bias due to confounding will be used for the meta-analysis [28]. If a study reported multiple PROMs under one construct, our review will prioritize cardiac-specific/stroke-specific PROMs over generic ones, as the former outcomes are more sensitive to the distinctive issues experienced by patients who have had an AMI or stroke. The heterogeneity of the studies will be measured with I^2^ statistics (I^2^ values of <25%, 25%-50%, 51%-75%,  and >75% indicate low, moderate, medium, and high heterogeneity, respectively), which describe the variation of effect size that is attributable to heterogeneity across studies [29]. Visual assessment of the funnel plot and Egger’s test will be used to assess for publication bias [30].

For studies that we are unable to meta-analyze, we will instead conduct a narrative synthesis. A summary table specifying study characteristics, outcomes, and results will be created under each outcome domain. The Grading of Recommendations Assessment, Development, and Evaluation (GRADE) system [31] will be used to evaluate the strength of the evidence body.

Subgroup analyses will be performed according to sex. To investigate the sources of heterogeneity, meta-regression will be performed adjusting for the covariates, including the duration of follow-up, study setting (hospital-based vs. population-based), bias score, mean age, male proportion, and sample size. If there is high heterogeneity, we will further conduct subgroup analyses according to the significant covariates. Sensitivity analysis will be performed by excluding studies with “low quality” according to the Newcastle-Ottawa Scale and comparing the results with the overall analysis. All quantitative analyses will be conducted in R using the meta library.

### Ethics and dissemination

This study is exempt from institutional review board approval because it is a secondary analysis of published or aggregated data. No new patients will be involved in this study. The data collected during the review will be made fully available without restriction upon study completion.

## Discussion

AMI and stroke represent a considerable population disease burden and are leading causes of death and disability worldwide [32, 33]. Although advances in prevention, diagnosis, and therapeutics led to increasing numbers of people surviving AMI and stroke, these diseases impact many aspects of the lives of affected individuals following hospital discharge [33].

A better prognosis after AMI and stroke among those who are married or have partners is supported by studies varying in design, setting, and scale [1–6]. A meta-analysis of 34 studies found 42% higher odds of mortality (95% CI: 1.14–1.76) for unmarried AMI patients compared to their married counterparts [1]. Another review provided evidence on the association of marriage with longer event-free survival and better risk factor control among cardiovascular patients [4]. At least one study reported poorer outcomes after stroke for those who were not married [34]. Despite existing review articles focusing on mortality and morbidity outcomes, there remains a lack of understanding of the impact of marital/partner status on patient-centered outcomes measured by PROMs.

PROMs can be considered an umbrella term for self-rating instruments that measure constructs such as health, physical and mental well-being, HRQoL, and symptoms, among other items [8, 35] and are demonstrated to be an essential aspect of value-based healthcare for both cardiovascular disease and stroke [9, 11]. Incorporating values important to AMI and stroke patients into research, and consequently into clinical practice, carries the potential of moving healthcare towards more patient-centered care, however, one major challenge to PROM-related research is the heterogeneity. In the context of the current review topic, observational studies of patients with AMI and stroke assessing the associations between marital/partner status and patient-reported health status outcomes varied in study population, PROMs used to measure health status, and period of data collection relative to the index event. The results of these studies are inconsistent. For example, three studies found that compared to being single, being married was associated with better quality of life among those who had an AMI or stroke [14–16], but another study found no association [13]. By systematically reviewing the literature and assessing the available evidence, we aim to clarify and quantify the association of marital/partner status with patient-centered outcomes measured using PROMs and explore differences by sex. A better understanding of this gap in knowledge can inform the design of secondary prevention interventions to improve long-term recovery of those who had an AMI or stroke and potentially support targeted interventions.

This systematic review will face several challenges. First, although studies with proxy responses are eligible for inclusion in the review, patients who have specific stroke-related deficits such as aphasia or cognitive impairments may be excluded from some studies according to their prespecified criteria, introducing potential bias. Second, we will conduct our literature search using multiple databases, but articles published in languages other than English will not be eligible. Third, because we will consider a variety of study designs and settings with a wide spectrum of PROMs, heterogeneity between studies is anticipated to be high. We pre-specified an outcome framework of four domains and corresponding constructs, and we will classify and analyze PROMs within the same domain. We also plan to extract a set of study characteristics and conduct meta-regression to investigate the sources of heterogeneity if relevant data are available. Subgroup analysis will be performed based on the significant covariates identified from the meta-regression.

## Supporting information

S1 Checklist PRISMA-P (Preferred Reporting Items for Systematic review and Meta-Analysis Protocols) 2015 checklist: Recommended items to address in a systematic review protocol. (DOCX)

S1 Table Search strategy for each database. (DOCX)

S2 Table Data extraction form. (DOCX)
