# Supplementary material for: The association of marital/partner status with patient-reported health outcomes following acute myocardial infarction or stroke: Protocol for a systematic review and meta-analysis
Source: PLoS One. 2022 Nov 15;17(11):e0267771. doi: 10.1371/journal.pone.0267771 (PMC9665376; doi:10.1371/journal.pone.0267771)
Supplement: S1 Table — (DOCX) [file pone.0267771.s002.docx]

**S1 Table. Search strategy for each database**

| **Medline (via Ovid)**  **AMI**  <https://ovidsp.ovid.com/ovidweb.cgi?T=JS&NEWS=N&PAGE=main&SHAREDSEARCHID=3cMgEcBPhQNj2m9zHURc8LoBIYD5aQPEeI0x5TOEtgKNVapOqV44KeEmDdyFUmTVy> | 1 marital status/ or divorce/ or marriage/ or single person/ or widowhood/ or spouses/  2 (marital or spousal or romantic relationship* or unmarried or married or partnership* or marriage* or wife or wives or husband* or spouse* or domestic partner* or break-up or divorce* or widow* or spinster* or bachelor* or girlfriend* or boyfriend*).mp.  3 1 or 2  4 exp Myocardial Infarction/  5 exp acute coronary syndrome/  6 exp coronary disease/  7 (myocardial infarct* or acute coronary syndrome or coronary heart disease or isch?emic heart disease or heart attack* or AMI).mp.  8 4 or 5 or 6 or 7  9 (HR-PRO or HRPRO or HRQL or HRQoL or QL or QoL).ti,ab. or quality of life.mp. or (health index* or health indices or health profile*).ti,ab. or health status.mp. or ((patient or self or child or parent or carer or proxy) adj (appraisal* or appraised or report or reported or reporting or rated or rating* or based or assessed or assessment*)).ti,ab. or ((disability or function or functional or functions or subjective or utility or utilities or wellbeing or well being) adj2 (index or indices or instrument or instruments or measure or measures or questionnaire* or profile or profiles or scale or scales or score or scores or status or survey or surveys)).ti,ab.  10 exp patient reported outcome measures/  11 exp patient outcome assessment/  12 ("patient-reported outcome*" or (((self adj report*) or (patient adj2 (reported or centered or centred or preference* or experience* or perception or perceived))) and (measure* or questionnaire* or survey* or psychometric* or depress* or psychologic* or anxiety or symptom* or fatigue))).mp.  13 10 or 11 or 12  14 (EQ-5D or SF-12 or SF-36 or "general health questionnaire" or PROMIS).mp.  15 (seattle angina questionnaire or SAQ or Mac-New Questionnaire or HeartQoL or QLICD_CHD).mp.  16 14 or 15  17 9 or 13 or 16  18 3 and 8 and 17 |
| --- | --- |
| **Medline (via Ovid)**  **Stroke**  <https://ovidsp.ovid.com/ovidweb.cgi?T=JS&NEWS=N&PAGE=main&SHAREDSEARCHID=40SPLktNNc2CLmXbeUlVSP1wwfM3rrAZ5A98Smkjgi4WSQyNGAByqvmJHbGGuXcBQ> | 1 marital status/ or divorce/ or marriage/ or single person/ or widowhood/ or spouses/  2 (marital or spousal or romantic relationship* or unmarried or married or partnership* or marriage* or wife or wives or husband* or spouse* or domestic partner* or break-up or divorce* or widow* or spinster* or bachelor* or girlfriend* or boyfriend*).mp.  3 1 or 2  4 exp stroke/  5 cerebrovascular disorders/  6 (Stroke* or cerebrovascular disease* or cerebrovascular accident* or brain vascular accident*).mp.  7 4 or 5 or 6  8 (HR-PRO or HRPRO or HRQL or HRQoL or QL or QoL).ti,ab. or quality of life.mp. or (health index* or health indices or health profile*).ti,ab. or health status.mp. or ((patient or self or child or parent or carer or proxy) adj (appraisal* or appraised or report or reported or reporting or rated or rating* or based or assessed or assessment*)).ti,ab. or ((disability or function or functional or functions or subjective or utility or utilities or wellbeing or well being) adj2 (index or indices or instrument or instruments or measure or measures or questionnaire* or profile or profiles or scale or scales or score or scores or status or survey or surveys)).ti,ab.  9 exp patient reported outcome measures/  10 exp patient outcome assessment/  11 ("patient-reported outcome*" or (((self adj report*) or (patient adj2 (reported or centered or centred or preference* or experience* or perception or perceived))) and (measure* or questionnaire* or survey* or psychometric* or depress* or psychologic* or anxiety or symptom* or fatigue))).mp.  12 9 or 10 or 11  13 (EQ-5D or SF-12 or SF-36 or "general health questionnaire" or PROMIS).mp.  14 (Neuro-QOL or stroke impact scale or stroke specific qol or aphasia quality of life or SAQOL or stroke-adapted sickness impact profile or SA-SIP).mp.  15 13 or 14  16 8 or 12 or 15  17 3 and 7 and 16 |
| **Web of Science**  **AMI**  <https://www.webofscience.com/wos/woscc/summary/5c648275-3fd4-49e6-9701-d5c22579a0b3-43e77cb9/relevance/1> | 1 TS=("marital status" OR divorce OR marriage OR "single person" OR widowhood OR spouses)  2 TS=(marital OR spousal OR "romantic relationship*" OR unmarried OR married OR partnership* OR marriage* OR wife OR wives OR husband* OR spouse* OR "domestic partner*" OR break-up OR divorce* OR widow* OR spinster* OR bachelor* OR girlfriend* OR boyfriend*)  3 #2 OR #1  4 TS="myocardial infarction"  5 TS="myocardial infarct"  6 TS="acute coronary syndrome"  7 TS="coronary disease"  8 TS=("ischemic heart disease" OR "ischaemic heart disease" OR "heart attack*" OR AMI)  9 #4 OR #5 OR #6 OR #7 OR #8  10 TI=(HR-PRO or HRPRO or HRQL or HRQoL or QL or QoL or "health index*" or "health indices" or "health profile*") OR AB=(HR-PRO or HRPRO or HRQL or HRQoL or QL or QoL or "health index*" or "health indices" or "health profile*") OR TS=("quality of life" OR "health status") OR TI= ((patient or self or child or parent or carer or proxy) NEAR (appraisal* or appraised or report or reported or reporting or rated or rating* or based or assessed or assessment*)) OR AB= ((patient OR self or child or parent or carer or proxy) NEAR (appraisal* or appraised or report or reported or reporting or rated or rating* or based or assessed or assessment*)) OR TI=((disability or function or functional or functions or subjective or utility or utilities or wellbeing or "well being") NEAR (index or indices or instrument or instruments or measure or measures or questionnaire* or profile or profiles or scale or scales or score or scores or status or survey or surveys)) OR AB=((disability or function or functional or functions or subjective or utility or utilities or wellbeing or "well being") NEAR (index or indices or instrument or instruments or measure or measures or questionnaire* or profile or profiles or scale or scales or score or scores or status or survey or surveys))  11 TS="patient reported outcome measures"  12 TS="patient outcome assessment"  13 TS=("patient-reported outcome*" OR ((patient OR self) AND report* AND (outcome* OR measure* OR questionnaire* OR survey* OR psychometric*)))  14 #11 or #12 or #13  15 ALL=(EQ-5D OR SF-36 OR SF-12 OR "general health questionnaire" OR PROMIS)  16 ALL=("seattle angina questionnaire" OR SAQ OR "Mac-New Questionnaire" OR HeartQoL OR QLICD_CHD)  17 #15 or #16  18 #10 or #14 or #17  19 #3 and #9 and #18 |
| **Web of Science**  **Stroke**  <https://www.webofscience.com/wos/woscc/summary/f12ed73d-f796-4817-9e06-88e937801c1f-43e7e6d3/relevance/1> | 1 TS=("marital status" OR divorce OR marriage OR "single person" OR widowhood OR spouses)  2 TS=(marital OR spousal OR "romantic relationship*" OR unmarried OR married OR partnership* OR marriage* OR wife OR wives OR husband* OR spouse* OR "domestic partner*" OR break-up OR divorce* OR widow* OR spinster* OR bachelor* OR girlfriend* OR boyfriend*)  3 #2 OR #1  4 TS="cerebrovascular disorders"  5 TS=stroke  6 TS=("cerebrovascular disease*" OR "cerebrovascular accident*" OR “brain vascular accident*”)  7 #4 or #5 or #6  8 TI=(HR-PRO or HRPRO or HRQL or HRQoL or QL or QoL or "health index*" or "health indices" or "health profile*") OR AB=(HR-PRO or HRPRO or HRQL or HRQoL or QL or QoL or "health index*" or "health indices" or "health profile*") OR TS=("quality of life" OR "health status") OR TI= ((patient or self or child or parent or carer or proxy) NEAR (appraisal* or appraised or report or reported or reporting or rated or rating* or based or assessed or assessment*)) OR AB= ((patient OR self or child or parent or carer or proxy) NEAR (appraisal* or appraised or report or reported or reporting or rated or rating* or based or assessed or assessment*)) OR TI=((disability or function or functional or functions or subjective or utility or utilities or wellbeing or "well being") NEAR (index or indices or instrument or instruments or measure or measures or questionnaire* or profile or profiles or scale or scales or score or scores or status or survey or surveys)) OR AB=((disability or function or functional or functions or subjective or utility or utilities or wellbeing or "well being") NEAR (index or indices or instrument or instruments or measure or measures or questionnaire* or profile or profiles or scale or scales or score or scores or status or survey or surveys))  9 TS="patient reported outcome measures"  10 TS="patient outcome assessment"  11 TS=("patient-reported outcome*" OR ((patient OR self) AND report* AND (outcome* OR measure* OR questionnaire* OR survey* OR psychometric*)))  12 #9 or #10 or #11  13 ALL=(EQ-5D OR SF-36 OR SF-12 OR "general health questionnaire" OR PROMIS)  14 ALL=(Neuro-QOL OR "stroke impact scale" OR "stroke specific qol" OR "aphasia quality of life" OR SAQOL OR "stroke-adapted sickness impact profile" OR SA-SIP)  15 #13 or #14  16 #8 or #12 or #15  19 #3 and #7 and #16 |
| **Scopus**  **AMI** | ( INDEXTERMS ( "marital status" ) OR INDEXTERMS ( divorce ) OR INDEXTERMS ( marriage ) OR INDEXTERMS ( "single person" ) OR INDEXTERMS ( widowhood ) OR INDEXTERMS ( spouses ) OR TITLE-ABS-KEY ( marital OR spousal OR "romantic relationship*" OR unmarried OR married OR partnership* OR marriage* OR wife OR wives OR husband* OR spouse* OR "domestic partner*" OR break-up OR divorce* OR widow* OR spinster* OR bachelor* OR girlfriend* OR boyfriend* ) ) AND ( INDEXTERMS ( "Myocardial Infarct*" ) OR INDEXTERMS ( "acute coronary syndrome" ) OR INDEXTERMS ( "coronary disease" ) OR TITLE-ABS-KEY ( "myocardial infarction" OR "acute coronary syndrome" OR "coronary heart disease" OR "isch?emic heart disease" OR "heart attack*" OR ami ) ) AND ( ALL ( "patient-reported outcome*" ) OR TITLE-ABS-KEY ( ( ( self W/1 report* ) OR ( patient W/2 ( reported OR centered OR centred OR preference* OR experience* OR perception OR perceived ) ) AND ( measure* OR questionnaire* OR survey* OR psychometric* OR depress* OR psychologic* OR anxiety OR symptom* OR fatigue ) ) ) OR INDEXTERMS ( "patient reported outcome measures" ) OR INDEXTERMS ( "patient outcome assessment" ) OR TITLE-ABS-KEY ( eq-5d OR sf-12 OR sf-36 OR "general health questionnaire" OR promis ) OR TITLE-ABS-KEY ( "seattle angina questionnaire" OR saq OR "Mac-New Questionnaire" OR heartqol OR qlicd_chd ) OR TITLE-ABS ( hr-pro OR hrpro OR hrql OR hrqol OR ql OR qol ) OR TITLE-ABS-KEY ( "quality of life" ) OR TITLE-ABS ( "health index*" OR "health indices" OR "health profile*" ) OR TITLE-ABS-KEY ( "health status" ) OR TITLE-ABS ( ( patient OR self OR child OR parent OR carer OR proxy ) W/1 ( appraisal* OR appraised OR report OR reported OR reporting OR rated OR rating* OR based OR assessed OR assessment* ) ) OR TITLE-ABS ( ( disability OR function OR functional OR functions OR subjective OR utility OR utilities OR wellbeing OR "well being" ) W/2 ( index OR indices OR instrument OR instruments OR measure OR measures OR questionnaire* OR profile OR profiles OR scale OR scales OR score OR scores OR status OR survey OR surveys ) ) ) |
| **Scopus**  **Stroke** | ( INDEXTERMS ( "marital status" ) OR INDEXTERMS ( divorce ) OR INDEXTERMS ( marriage ) OR INDEXTERMS ( "single person" ) OR INDEXTERMS ( widowhood ) OR INDEXTERMS ( spouses ) OR TITLE-ABS-KEY ( marital OR spousal OR "romantic relationship*" OR unmarried OR married OR partnership* OR marriage* OR wife OR wives OR husband* OR spouse* OR "domestic partner*" OR break-up OR divorce* OR widow* OR spinster* OR bachelor* OR girlfriend* OR boyfriend* ) ) AND ( INDEXTERMS ( "cerebrovascular disorders" ) OR INDEXTERMS ( stroke ) OR TITLE-ABS-KEY ( stroke* OR "cerebrovascular disease*" OR "cerebrovascular accident*" OR "brain vascular accident*" ) ) AND ( ALL ( "patient-reported outcome*" ) OR TITLE-ABS-KEY ( ( ( self W/1 report* ) OR ( patient W/2 ( reported OR centered OR centred OR preference* OR experience* OR perception OR perceived ) ) AND ( measure* OR questionnaire* OR survey* OR psychometric* OR depress* OR psychologic* OR anxiety OR symptom* OR fatigue ) ) ) OR INDEXTERMS ( "patient reported outcome measures" ) OR INDEXTERMS ( "patient outcome assessment" ) OR TITLE-ABS-KEY ( eq-5d OR sf-12 OR sf-36 OR "general health questionnaire" OR promis ) OR TITLE-ABS-KEY ( neuro-qol OR "stroke impact scale" OR "stroke specific qol" OR "aphasia quality of life" OR saqol OR "stroke-adapted sickness impact profile" OR sa-sip ) OR TITLE-ABS ( hr-pro OR hrpro OR hrql OR hrqol OR ql OR qol ) OR TITLE-ABS-KEY ( "quality of life" ) OR TITLE-ABS ( "health index*" OR "health indices" OR "health profile*" ) OR TITLE-ABS-KEY ( "health status" ) OR TITLE-ABS ( ( patient OR self OR child OR parent OR carer OR proxy ) W/1 ( appraisal* OR appraised OR report OR reported OR reporting OR rated OR rating* OR based OR assessed OR assessment* ) ) OR TITLE-ABS ( ( disability OR function OR functional OR functions OR subjective OR utility OR utilities OR wellbeing OR "well being" ) W/2 ( index OR indices OR instrument OR instruments OR measure OR measures OR questionnaire* OR profile OR profiles OR scale OR scales OR score OR scores OR status OR survey OR surveys ) ) ) |
| **EMBASE (via Ovid)**  **AMI**  <https://ovidsp.ovid.com/ovidweb.cgi?T=JS&NEWS=N&PAGE=main&SHAREDSEARCHID=gQZohWwsHAOKtBH5lZY2R6g2AsNMBkKZOvVw62o64ZrQAd9wBGYoKDrnMiYxj7bE> | 1 marriage/ or "single person"/ or exp widowed person/ or spouse/  2 (marital or spousal or "romantic relationship*" or unmarried or married or partnership* or marriage* or wife or wives or husband* or spouse* or "domestic partner*" or break-up or divorce* or widow* or spinster* or bachelor* or girlfriend* or boyfriend*).mp.  3 1 or 2  4 heart Infarction/  5 acute coronary syndrome/  6 coronary artery disease/  7 (“myocardial infarct*” or “acute coronary syndrome” or “coronary heart disease” or “isch?emic heart disease” or “heart attack*” or AMI).mp.  8 4 or 5 or 6 or 7  9 (HR-PRO or HRPRO or HRQL or HRQoL or QL or QoL).ti,ab. or quality of life.mp. or (health index* or health indices or health profile*).ti,ab. or health status.mp. or ((patient or self or child or parent or carer or proxy) adj (appraisal* or appraised or report or reported or reporting or rated or rating* or based or assessed or assessment*)).ti,ab. or ((disability or function or functional or functions or subjective or utility or utilities or wellbeing or well being) adj2 (index or indices or instrument or instruments or measure or measures or questionnaire* or profile or profiles or scale or scales or score or scores or status or survey or surveys)).ti,ab.  10 patient-reported outcome/  11 outcome assessment/  12 ("patient-reported outcome*" or (((self adj report*) or (patient adj2 (reported or centered or centred or preference* or experience* or perception or perceived))) and (measure* or questionnaire* or survey* or psychometric* or depress* or psychologic* or anxiety or symptom* or fatigue))).mp.  13 10 or 11 or 12  14 (EQ-5D or SF-12 or SF-36 or "general health questionnaire" or PROMIS).mp.  15 ("seattle angina questionnaire" or SAQ or "Mac-New Questionnaire" or HeartQoL or QLICD_CHD).mp.  16 14 or 15  17 9 or 13 or 16  18 3 and 8 and 17 |
| **EMBASE (via Ovid)**  **Stroke**  <https://ovidsp.ovid.com/ovidweb.cgi?T=JS&NEWS=N&PAGE=main&SHAREDSEARCHID=63eKRHNejZzpF3QfOY3vwB1cARlEtn8ce61njozKZuzRjuDsbEaqE3hga23AayUwz> | 1 marriage/ or "single person"/ or exp widowed person/ or spouse/  2 (marital or spousal or "romantic relationship*" or unmarried or married or partnership* or marriage* or wife or wives or husband* or spouse* or "domestic partner*" or break-up or divorce* or widow* or spinster* or bachelor* or girlfriend* or boyfriend*).mp.  3 1 or 2  4 cerebrovascular disease/  5 (Stroke* or "cerebrovascular disease*" or "cerebrovascular accident*" or "brain vascular accident*").mp.  6 4 or 5  7 (HR-PRO or HRPRO or HRQL or HRQoL or QL or QoL).ti,ab. or quality of life.mp. or (health index* or health indices or health profile*).ti,ab. or health status.mp. or ((patient or self or child or parent or carer or proxy) adj (appraisal* or appraised or report or reported or reporting or rated or rating* or based or assessed or assessment*)).ti,ab. or ((disability or function or functional or functions or subjective or utility or utilities or wellbeing or well being) adj2 (index or indices or instrument or instruments or measure or measures or questionnaire* or profile or profiles or scale or scales or score or scores or status or survey or surveys)).ti,ab.  8 patient-reported outcome/  9 outcome assessment/  10 ("patient-reported outcome*" or (((self adj report*) or (patient adj2 (reported or centered or centred or preference* or experience* or perception or perceived))) and (measure* or questionnaire* or survey* or psychometric* or depress* or psychologic* or anxiety or symptom* or fatigue))).mp.  11 8 or 9 or 10  12 (EQ-5D or SF-12 or SF-36 or "general health questionnaire" or PROMIS).mp.  13 (Neuro-QOL or "stroke impact scale" or "stroke specific qol" or "aphasia quality of life" or SAQOL or "stroke-adapted sickness impact profile" or SA-SIP).mp.  14 12 or 13  15 7 or 11 or 14  16 3 and 6 and 15 |
| **PsycINFO (via Ovid)**  **AMI**  <https://ovidsp.ovid.com/ovidweb.cgi?T=JS&NEWS=N&PAGE=main&SHAREDSEARCHID=59VLQqpRfxNetKok4QhQrkVB4ydAy3kpeVxSJKkewKtdqFcpcJZ46Delk8x74q6nO> | 1 marital status/ or "marital separation"/ or divorce/ or marriage/ or "single persons"/ or widowers/ or widows.mp. or exp spouses/ or "marital separation"/ or "significant others"/ [mp=title, abstract, heading word, table of contents, key concepts, original title, tests & measures, mesh word]  2 (marital or spousal or "romantic relationship*" or unmarried or married or partnership* or marriage* or wife or wives or husband* or spouse* or "domestic partner*" or break-up or divorce* or widow* or spinster* or bachelor* or girlfriend* or boyfriend*).mp.  3 1 or 2  4 exp "Myocardial Infarctions"/  5 ("myocardial infarct*" or "acute coronary syndrome" or "coronary heart disease" or "isch?emic heart disease" or "heart attack*" or AMI).mp.  6 4 or 5  7 (HR-PRO or HRPRO or HRQL or HRQoL or QL or QoL).ti,ab. or quality of life.mp. or (health index* or health indices or health profile*).ti,ab. or health status.mp. or ((patient or self or child or parent or carer or proxy) adj (appraisal* or appraised or report or reported or reporting or rated or rating* or based or assessed or assessment*)).ti,ab. or ((disability or function or functional or functions or subjective or utility or utilities or wellbeing or well being) adj2 (index or indices or instrument or instruments or measure or measures or questionnaire* or profile or profiles or scale or scales or score or scores or status or survey or surveys)).ti,ab.  8 exp "patient reported outcome measures"/  9 ("patient-reported outcome*" or (((self adj report*) or (patient adj2 (reported or centered or centred or preference* or experience* or perception or perceived))) and (measure* or questionnaire* or survey* or psychometric* or depress* or psychologic* or anxiety or symptom* or fatigue))).mp.  10 8 or 9  11 (EQ-5D or SF-12 or SF-36 or "general health questionnaire" or PROMIS).mp.  12 ("seattle angina questionnaire" or SAQ or "Mac-New Questionnaire" or HeartQoL or QLICD_CHD).mp.  13 11 or 12  14 7 or 10 or 13  15 3 and 6 and 14 |
| **PsycINFO (via Ovid)**  **Stroke**  <https://ovidsp.ovid.com/ovidweb.cgi?T=JS&NEWS=N&PAGE=main&SHAREDSEARCHID=5jFr6QdVj7y7BxdNXIvk4xdjYufSU1dGYihG8r1iEXSqjrawI7ESYvcGLSx2uHnTN> | 1 marital status/ or "marital separation"/ or divorce/ or marriage/ or "single persons"/ or widowers/ or widows.mp. or exp spouses/ or "marital separation"/ or "significant others"/ [mp=title, abstract, heading word, table of contents, key concepts, original title, tests & measures, mesh word]  2 (marital or spousal or "romantic relationship*" or unmarried or married or partnership* or marriage* or wife or wives or husband* or spouse* or "domestic partner*" or break-up or divorce* or widow* or spinster* or bachelor* or girlfriend* or boyfriend*).mp.  3 1 or 2  4 "cerebrovascular disorders"/  5 "cerebrovascular accidents"/  6 (Stroke* or "cerebrovascular disease*" or "cerebrovascular accident*" or "brain vascular accident*").mp.  7 4 or 5 or 6  8 (HR-PRO or HRPRO or HRQL or HRQoL or QL or QoL).ti,ab. or quality of life.mp. or (health index* or health indices or health profile*).ti,ab. or health status.mp. or ((patient or self or child or parent or carer or proxy) adj (appraisal* or appraised or report or reported or reporting or rated or rating* or based or assessed or assessment*)).ti,ab. or ((disability or function or functional or functions or subjective or utility or utilities or wellbeing or well being) adj2 (index or indices or instrument or instruments or measure or measures or questionnaire* or profile or profiles or scale or scales or score or scores or status or survey or surveys)).ti,ab.  9 exp "patient reported outcome measures"/  10 ("patient-reported outcome*" or (((self adj report*) or (patient adj2 (reported or centered or centred or preference* or experience* or perception or perceived))) and (measure* or questionnaire* or survey* or psychometric* or depress* or psychologic* or anxiety or symptom* or fatigue))).mp.  11 9 or 10  12 (EQ-5D or SF-12 or SF-36 or "general health questionnaire" or PROMIS).mp.  13 (Neuro-QOL or "stroke impact scale" or "stroke specific qol" or "aphasia quality of life" or SAQOL or "stroke-adapted sickness impact profile" or SA-SIP).mp.  14 12 or 13  15 8 or 11 or 14  16 3 and 7 and 15 |
