# Supplementary material for: The association of marital/partner status with patient-reported health outcomes following acute myocardial infarction or stroke: Protocol for a systematic review and meta-analysis
Source: PLoS One. 2022 Nov 15;17(11):e0267771. doi: 10.1371/journal.pone.0267771 (PMC9665376; doi:10.1371/journal.pone.0267771)
Supplement: S2 Table — (DOCX) [file pone.0267771.s003.docx]

**Table S2 – Data extraction form**

| **Reviewer:** | **Date:** |  |
| --- | --- | --- |
| **Study characteristics** | | |
| Author last name |  |  |
| Publication year | Country |  |
| Study type |  |  |
| ( ) Cohort | ( ) Case control | ( ) Cross sectional |
| Setting | ( ) Hospital-based | ( ) Population-based |
| Condition | ( ) AMI | ( ) Stroke |
| **Participants (individual/proxy/other)** | | |
| Sample size | Age (mean) | Sex: Male (n;%) Female (n;%) |
| Other notes |  |  |
| **Exposure of interest** | | |
| Definition of marital/partner status | ( ) Self-report | ( ) Other |
| Reference group (categories) |  |  |
| Other notes |  |  |
| **Outcomes** |  |  |
| PROM name |  |  |
| Definition |  |  |
| Domain measured |  |  |
| Measure time |  |  |
| Score in each group |  |  |
| Other notes |  |  |
| **Results** |  |  |
| # Participants with outcome |  |  |
| Mean score for each exposure group | | |
| Association type (OR/RR/HR/Other:) | | |
| Unadjusted/Adjusted association |  |  |
| Covariates adjusted |  |  |
| Sex-specific results (if any) |  |  |
| Other notes |  |  |
| **Author’s conclusion:** |  |  |
| **Reviewer comments:** |  |  |
